# The Clinical Impact of Time-restricted Eating on Cancer: A Systematic Review

**DOI:** 10.1093/nutrit/nuae105

**Published:** 2024-08-30

**Authors:** Eleah J Stringer, Rob W G Cloke, Lindsay Van der Meer, Rachel A Murphy, Nicol A Macpherson, Julian J Lum

**Affiliations:** Nursing and Allied Health Research and KT Department, BC Cancer, Vancouver, BC V5Z 1G1, Canada; Department of Oncology Nutrition, BC Cancer, Victoria, BC V8R 6V5, Canada; Food, Nutrition and Health, Faculty of Land and Food Systems, University of British Columbia, Vancouver, BC V6T 1Z4, Canada; Nursing and Allied Health Research and KT Department, BC Cancer, Vancouver, BC V5Z 1G1, Canada; Department of Microbiology and Immunology, University of British Columbia, Vancouver, BC V6T 1Z4, Canada; Nursing and Allied Health Research and KT Department, BC Cancer, Vancouver, BC V5Z 1G1, Canada; Department of Oncology Nutrition, BC Cancer, Victoria, BC V8R 6V5, Canada; Food, Nutrition and Health, Faculty of Land and Food Systems, University of British Columbia, Vancouver, BC V6T 1Z4, Canada; School of Population and Public Health, University of British Columbia, Vancouver, BC V6T 1Z3, Canada; Department of Cancer Control Research, BC Cancer Research Institute, Vancouver, BC V5Z 1L3, Canada; Department of Medical Oncology, BC Cancer – Victoria, Victoria, BC V8R 6V5, Canada; Department of Medical Oncology, Faculty of Medicine, University of British Columbia, Vancouver, BC V5Z 1M9, Canada; Trev and Joyce Deeley Research Centre, BC Cancer – Victoria, Victoria, BC V8R 6V5, Canada; Department of Biochemistry and Microbiology, University of Victoria, Victoria, BC V8W 2Y2, Canada

**Keywords:** intermittent fasting, time-restricted eating, dietary interventions, cancer, systematic review

## Abstract

**Context:**

In the face of the growing global burden of cancer, there is increasing interest in dietary interventions to mitigate its impacts. Pre-clinical evidence suggests that time-restricted eating (TRE), a type of intermittent fasting, induces metabolic effects and alterations in the gut microbiome that may impede carcinogenesis. Research on TRE in cancer has progressed to human studies, but the evidence has yet to be synthesized.

**Objective:**

The objective of this study was to systematically evaluate the clinical and/or metabolomic effects of TRE compared with ad libitum eating or alternative diets in people with cancer.

**Data Sources:**

Ovid MEDLINE, Ovid Embase, CINAHL, Ovid Cochrane Central Register of Control Trials (CENTRAL), Web of Science Core Collection (ESCI, CPCI-SSH, CPCI-S), and SCOPUS were searched up to January 4, 2023, using the core concepts of “intermittent fasting” and “cancer.” Original study designs, protocols, and clinical trial registries were included.

**Data Extraction:**

After evaluating 13 900 results, 24 entries were included, consisting of 8 full articles, 2 abstracts, 1 published protocol and 13 trial registries. All data were extracted, compared, and critically analyzed.

**Data Analysis:**

There was heterogeneity in the patient population (eg, in tumor sites), TRE regimens (eg, degree of restriction, duration), and clinical end points. A high rate (67–98%) of TRE adherence was observed, alongside improvements in quality of life. Four articles assessed cancer markers and found a reduction in tumor marker carcinoembryonic antigen, reduced rates of recurrence, and a sustained major molecular response, following TRE. Five articles demonstrated modified cancer risk factors, including beneficial effects on body mass index, adiposity, glucoregulation, and inflammation in as short a period as 8 weeks. None of the completed studies assessed the effect of TRE on the microbiome, but analysis of the microbiome is a planned outcome in 2 clinical trials.

**Conclusions:**

Preliminary findings suggest that TRE is feasible and acceptable by people with cancer, may have oncological benefits, and improves quality of life.

**Registration:**

PROSPERO registration No. CRD42023386885.

## INTRODUCTION

Cancer is the leading cause of death worldwide, with the global cancer burden expected to increase by 47% between 2020 and 2040.^1^ The relative magnitude of this increase is most striking in countries categorized as having a low or medium Human Development Index,^1^ emphasizing the need for equitable approaches for cancer control that are not resource-dependent. Dietary intake and interventions have been studied for decades in an effort to understand how diet may contribute to cancer control. Population-level, evidence-based dietary recommendations for the prevention of primary cancer and its recurrence consist of dietary guidelines that provide guidance on the type and amount of foods/nutrients,^2^ but little is known about whether the timing of food intake may impact cancer control.

Intermittent fasting (IF) is a dietary pattern alternating between periods of free eating and abstaining from food.^3^^,^^4^ There are a variety of IF regimens, including, short-term fasting, the fasting-mimicking diet, and periodic fasting, which may or may not also include a degree of caloric restriction.^5^^,^^6^ A lack of standardized terminology and implementation in this field muddles the literature, as terms are used inconsistently and, sometimes, interchangeably.^7^

**Figure 1.**
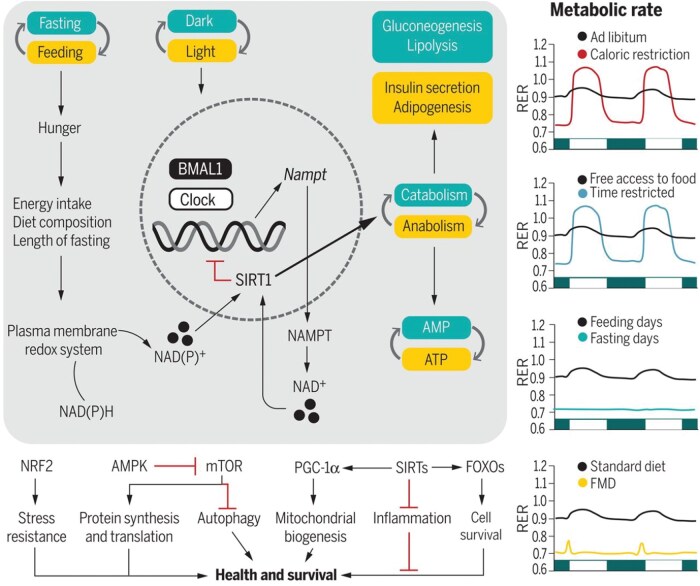
“Integration of the Circadian Rhythms and Feeding–Fasting Cycles with Metabolism.”^12^ The Respiratory Exchange Ratio (RER) is defined as the ratio between the amount of carbon dioxide produced and oxygen used during breathing

Time-restricted eating (TRE) is a form of IF that follows a rhythmic eating pattern that limits eating hours to a set window on a daily, or near-daily, basis.^8^^,^^9^ The eating window often aligns with the circadian rhythm (ie, eating during active daytime hours) to optimize metabolic health.^10^^,^^11^  Figure 1 demonstrates how TRE and daylight increase concentrations of the plasma membrane redox system enzymes and the production of nicotinamide adenine dinucleotide (NAD+), respectively, which are required to activate sirtuin 1 (SIRT-1). SIRT-1 then promotes the catabolism of glucose and inhibits the circadian clock machinery, impacting downstream transcription factors related to health, such as stress resistance, protein synthesis, and the translation of mRNA into protein.^12^

TRE contains inherent flexibility that allows individuals to maintain their food preferences, as there are no guidelines on which foods should be consumed.^9^^,^^13^ Some religious fasts, such as Ramadan fasting, can also be conceptualized as TRE, since intake is restricted to set hours on a daily basis. Unique to the holy month of Ramadan, the ninth month of the lunar year, fasting Muslims refrain from eating and drinking from dawn until sunset, which is in opposition to the circadian rhythm.^14^^,^^15^ In TRE, the eating window is frequently set at between 6 and 12 hours, as a minimum of 12 hours is needed to achieve a 20% or greater decrease in serum glucose, along with hepatic glycogen depletion.^5^^,^^16^ This necessitates a metabolic adaptation to the use of nonhepatic glucose, fat-derived ketone bodies, and free fatty acids as energy sources.^12^ This metabolic switch is illustrated in the right-hand column of Figure 1, showing the respiratory exchange ratio (RER) oscillating between 0.7 and 1.1 with the higher RER indicating utilization of carbohydrates and the lower RER signaling the use of lipids.^12^ Physiologically, the body acclimates by restoring metabolic regulators, increasing insulin sensitivity and cellular stress resistance, and reducing diastolic blood pressure and heart rate, all of which may contribute to the health benefits of IF.^17^^,^^18^

Furthermore, studies suggest that the effects of IF on metabolism may be closely associated with alterations in the gut microbiota.^19–22^ Altering the microbiome and microbiota-derived metabolites can influence the cancer process through a variety of mechanisms, including cell differentiation, apoptosis, immunity, and hormonal regulation.^23^^,^^24^ Treatment with chemotherapy, radiation, and/or antibiotics disrupts the composition of the gut microbiome, and can lead to dysbiosis,^25^ which is thought to shape responses to treatment and impact the toxicity severity. In addition to mitigating the effects of chemotherapy and radiation, the gut microbiota and microbiota-derived metabolites may alter the efficacy and toxicity of immunotherapies.^26^^,^^27^ Multiple studies have found differences in the taxonomic composition of the gut microbiota between patients who respond to checkpoint inhibitors compared with non-responders.^27–32^ TRE may help restore and/or maintain a healthy microbiome. Ramadan fasting, for example, has been demonstrated to increase the abundance of *Akkermansia muciniphila, Bacteroides fragilis, Bacteroides* sp. and butyric acid–producing Lachnospiraceae, which are all demonstrated to be key members of a healthy microbiome.^21^^,^^33^^,^^34^

Recent research has demonstrated TRE exerting tumor-suppressing effects in healthy individuals. For example, Jamshed et al (2019) tested TRE (18-hour fasting per day), through a randomized crossover study, in 11 overweight but otherwise healthy adults and, relative to the control group, found capillary glucose decreased by 4 ± 1 mg/dL (*P* = .0003) and glycemic excursions decreased by 12 ± 3 mg/dL (*P* = .001). When overnight fasting extended into the morning, TRE resulted in statistically significant increases in ketones, cholesterol, and the expression of genes *LC3A* and *SIRT-1*, related to autophagy, and oxidative stress and aging, respectively. During the night, TRE increased brain-derived neurotropic factor and increased the expression of *MTOR*, suggesting that TRE improves glucose levels, alters lipid metabolism and circadian clock gene expression, and may increase autophagy in humans.^34^ Mindikoglu et al (2020) demonstrated in 14 healthy individuals that 30-day TRE (minimum 14 hour fasting per day) was associated with an anticancer serum proteome response and an upregulation of several key regulatory proteins that play a crucial role in tumor suppression, DNA repair, insulin signaling, glucose and lipid metabolism, circadian clock function, cytoskeletal remodeling, immune system function, and cognitive function. Importantly, the increase in these critical regulatory proteins occurred in the absence of any significant weight loss or calorie restriction, which is an important consideration for people with cancer who have underlying or treatment-induced metabolic decompensation.^35^

While the preliminary clinical research suggests that TRE is promising as a cancer prevention strategy in healthy populations, less is known about the effects of TRE in people with cancer. Here, we conducted a systematic literature review to evaluate the clinical and metabolomic effects of TRE, compared with ad libitum eating or alternative diets, in people with cancer.

## METHODS

The 5-step methodology for health research systematic reviews, described by Khan et al (2003) was followed^36^ and conducted in accordance with PRISMA guidelines.^37^ The protocol was registered at the International Prospective Register of Systematic Reviews (PROSPERO) registry (CRD No. 42023386885) on February 11, 2023.

### Eligibility criteria

The eligibility criteria were designed to address the study objective and are summarized in Table 1. Clinical trial registries and abstracts were only included if the results or full article were not yet available. Articles were required to include data on at least 1 outcome of interest, including clinical (eg, mortality statistics), biochemical, anthropometric, or quality of life (QOL) measures; metabolomics; or the microbiome; ethnographies were excluded.

**Table 1.** Article Inclusion and Exclusion Criteria in PICOS Format

Parameter	Inclusion criterion	Exclusion criterion
Participants	Human, oncology (any type and stage, with or without treatment), adult	Ex vivo; pediatric
Intervention	Diet focus on time-restricted feeding, such as 16/8 TRE, Ramadan, or minor variations (eg, 14/10 or 18/6) for any duration	Diet focus on other versions of intermittent feeding (eg, short-term or alternate-day fasting), preprocedural fasting (eg, ^18F^FDG) or perioperative fasting (eg, enhanced recovery after surgery)
Comparisons	Ad libitum eating, alternative diets (eg, caloric restriction)	N/A
Outcomes	Direct cancer outcome (eg, mortality); indirect cancer outcome (eg, quality of life); cancer risk factors (eg, BMI); microbiome/metabolomics	Nonclinical (eg, ethnographies), pharmacokinetics
Study designs	Single-arm interventional trials; non/randomized control trials, crossovers, cohort, case–control	Systematic reviews, scoping reviews, narratives reviews
Publication types	Academic, peer reviewed original research, gray literature (case studies, clinical trial registries only), protocol publications (if study is not yet completed), preprints (if complete, peer-reviewed version not yet published); English language	Protocols (if results published, then article included instead)

*Abbreviation:* N/A, not applicable.

### Information sources and search strategy

Ovid MEDLINE, Ovid Embase, CINAHL, the Ovid Cochrane Central Register of Control Trials (CENTRAL), the Web of Science Core Collection (ESCI, CPCI-SSH, CPCI-S), and SCOPUS were selected, based on their relevance to health research. The search strategy was developed around the core concepts of IF, cancer, and clinical trials (see Appendix SA). Databases were searched using MeSH explosions and Boolean operators, proximity operators, truncation, and wildcard symbols. Search strategies were developed in consultation with a research librarian specializing in human nutrition, piloted in each database, then further optimized. The indexing of the search limiters built into the various databases were researched, and limiters were tested prior to application. Search term “eating or feeding behaviours” was excluded from the final search strategy, as the results did not prove relevant to the inclusion/exclusion criteria and introduced significant noise. Gray literature, including trial registries, were searched through Google Scholar and clinicaltrials.gov. Reference lists were also searched to identify relevant articles. Searches were limited to English articles, but no limits were placed on the publication date. The search was conducted on January 4, 2023. The final search strategies are provided in Appendix SB.

### Selection process

Search results were imported into Covidence,^38^ and were reviewed by 4 team members. Articles were screened first by title and abstract, then by full text, independently by 2 reviewers, who applied the inclusion/exclusion criteria listed in Table 1. E.S. screened all articles for an additional layer of consistency. Conflicts were discussed and resolved through unanimous decision with a third reviewer.

### Data collection process and data item

Data was extracted independently by 2 reviewers in Covidence, then compared to verify the accuracy of extraction. Data items extracted included: article characteristics (country, study design, number of participants), cancer type (location and staging), fasting regimen (including duration) and comparison group, feasibility markers (recruitment, adherence/compliance, attrition rates, qualitative feedback), results/impact on cancer OR outcomes of interest from protocols (for active, incomplete studies), results/impact on microbiome or metabolomic (if included), themes across articles, recommended future areas of study, and bias. The effect measure corresponding to each result was recorded and used in the presentation of results.

### Synthesis methods

All extracted end points were categorized into either clinical or metabolic, with 3 clinical subcategories—cancer markers, risk factors, and QOL. Data was further stratified by article type. For protocols and trial registries, proposed outcome measures and methods of collection were extracted, since results were not yet available.

### Risk of bias assessment

All results underwent a risk-of-bias assessment, except for trial registries that were suspended or withdrawn. The assessment was conducted independently by team members E.S. and R.C., and conflicts were agreed upon through discussion. The Cochrane Risk-of-Bias tools, including Risk of Bias in Randomized Trials version 2 (RoB 2.0),^39^ Risk Of Bias In Non-randomized Studies—of Exposures (ROBINS-E),^40^ and Risk Of Bias In Non-randomized Studies—of Interventions (ROBINS-I)^41^ were used to assess randomized control trials (RCTs), crossover trials, nonrandomized trials, and cohort studies, respectively. The National Institute of Health’s Quality Assessment Tool for Before–After (Pre–Post) Studies With No Control Group^42^ was used to assess single-arm feasibility trials. The Cochrane robvis visualization tool^43^ was used to produce the stop light and color gradient visualizations of the results.

## RESULTS

### Study selection

**Figure 2.**
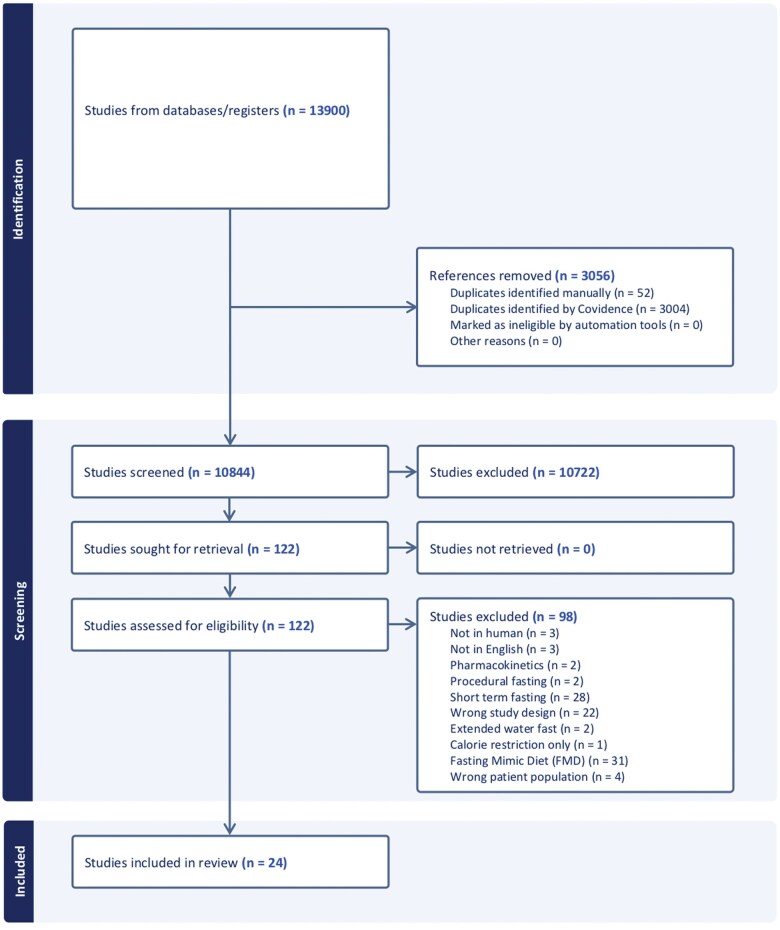
PRISMA Diagram

A total of 13 900 articles were retrieved. Duplicates were removed and 10 844 articles underwent title and abstract screening. A full article review was conducted on 122 articles, resulting in 24 articles being included for data extraction (the full reference list in Appendix SC). Sixty-four percent of the articles excluded at full-text review were excluded due to implementing an IF regimen other than TRE, such as the fasting-mimicking diet or short-term fasting near chemotherapy administration (see PRISMA diagram) (Figure 2).

### Study characteristics

The 24 included studies consisted of 89 full articles, 2 abstracts, 1 published protocol, and 13 clinical trial registries (Table 2). Four of the trial registries where discontinued or withdrawn. The most common study design was single-arm, pre/post feasibility trials, representing 33% (*n* = 8) of the results, followed next by RCTs (*n* = 5, 21%). The research locations were dominated by the United States (*n* = 15, 63%), followed by Canada (*n* = 4, 17%), the Middle East (*n* = 3, 13%), Spain (*n* = 1), and Chile (*n* = 1). Nearly half of the studies were on breast cancer (*n* = 11, 46%), followed by blood cancer (*n* = 3), prostate cancer (*n* = 2), colorectal cancer (*n* = 1), endometrial cancer (*n* = 1), and head and neck cancer (*n* = 1). Five studies (21%) included patients with 2 or more tumor sites, classified as “mixed” in Table 2.

**Table 2.** Study Characteristics, *n* = 24

Study	**Publication year** ^a^	Type	Design	**Study phase** (trial registries)	Country	Sample Size	Cancer type
Alshammari et al	2022	Full article	Prospective cohort	N/A	Saudi Arabia	37	Colorectal
Badar et al	2014	Full article	Crossover	N/A	Saudi Arabia	11	Mixed (2+ types)
Christensen et al	2022	Published protocol	Randomized controlled trial	Not listed, presumed phase II	Canada	130 (planned)	Breast
Kirkham et al	2021	Abstract	Crossover	N/A	Canada	15	Breast
Kirkham et al	2022	Full article	Crossover	N/A	Canada	22	Breast
Kleckner et al	2022	Full article	Single-arm feasibility	N/A	United States	39	Mixed (2+ types)
Marinac et al	2016	Full article	Retrospective cohort	N/A	United States	2413	Breast
O’Donnell et al	2022	Full article	Single-arm feasibility	N/A	United States	40	Breast
Palomar-Cros et al	2021	Full article	Retrospective case–control	N/A	Spain	1455 (607 cases, 848 controls)	Prostate
Vega et al	2022	Abstract	Nonrandomized experimental	N/A	Chile	16	Mixed (2+ types)
Yassin et al	2021	Full article	Retrospective cohort	N/A	Qatar	49	Blood
NCT03523377	2018	Trial Registry—active	Randomized controlled trial	N/A	United States	40	Mixed (2+ types)
NCT04288336	2020	Trial Registry—discontinued	Single-arm feasibility	Early phase I	United States	0	Prostate
NCT04560439	2020	Trial Registry—discontinued	Single-arm feasibility	N/A	United States	0	Breast
NCT04626843	2020	Trial Registry—active	Single-arm feasibility	Phase I	Canada	15	Blood
NCT04691999	2020	Trial Registry—discontinued	Single-arm feasibility	N/A	United States	0	Breast
NCT04708860	2021	Trial Registry—active	Single-arm feasibility	N/A	United States	30	Breast
NCT04783467	2021	Trial Registry—active	Crossover	N/A	United States	15 (estimated)	Endometrial
NCT05023967	2020	Trial Registry—active	Randomized controlled trial	Phase II	United States	120	Breast
NCT05083416	2021	Trial Registry—active	Non-randomized experimental	N/A	United States	29	Head and neck
NCT05259410	2022	Trial Registry—active	Randomized controlled trial	N/A	United States	40	Breast
NCT05312255	2022	Trial Registry—active	Non-randomized experimental	N/A	United States	150	Blood
NCT05327608	2022	Trial Registry—discontinued	Single-arm feasibility	Phase II	United States	55	Breast
NCT05722288	2023	Trial Registry—active	Randomized controlled trial	Phase II	United States	60	Mixed (2+ types)

a Clinical trial registration year used for trial registries. *Abbreviation*: N/A, not applicable.

Of the 10 articles, 3 reported on Ramadan fasting,^44–46^ while the remaining reported on nonreligious TRE (Table 3). Each of the studies analyzing Ramadan fasting used a different comparator group. The study by Alshammari and colleagues (2022) compared results between those who observed Ramadan fasting for 20 or more days against those who fasted for less than 20 days. Badar et al (2014) compared the last 2 weeks of Ramadan against the first 2 weeks of Shawwal (nonfasting), following a washout period of at least 2 weeks. Lastly, Yassin et al (2021) used within-person analysis to compare measures before and after observing Ramadan.

**Table 3.** Description of Fasting Interventions

Study	Description	Days/week	Duration	Adherence	Control/comparator
Alshammari et al, 2022	Ramadan: fasting from dawn to sunset	7	≥20 d	70%	<20 d
Badar et al, 2014	Ramadan: fasting from dawn to sunset	7	Last 2 wks of Ramadan		2 wks during Shawwal (nonfasting)
Kirkham et al, 2021	16/8 TRE on weekdays, eating ad libitum but only between 12 pm and 8 pm on weekdays or any time of day on weekends	5	8 wks	98% (range 93–100%)	N/A
Kirkham et al, 2022	16/8 TRE on weekdays, eating ad libitum but only between 12 pm and 8 pm on weekdays or any time of day on weekends; consumption of only water, black coffee, or black tea outside of those hours	5	8 wks	98% (range: 85–100%)	N/A
Kleckner et al, 2022	TRE of 10 h eating window during the day. Hours selected by the participant based on their normal meal patterns and preferences. Water and medications were allowable any time, but, coffee, tea, chewing gum, and diet beverages were discouraged during the fasting window.	7^a^	14 d	90%	N/A
Marinac et al, 2016	Nightly fasting duration of ≥13 h	7^a^	Nightly fasting duration was estimated at baseline, 1 y, and 4 y		Fasting for <13 h overnight
O’Donnell et al, 2022	Overnight fast of 13 h	7^a^	12 wks	93%	N/A
Palomar-Cros et al, 2021	Fasting >11 h per night	7^a^	Not reported		Control group fasting for <11 h overnight
Vega et al, 2022	Fasting window of ≥16 h	7^a^	12 wks	75%	Caloric restriction of 25% of basal caloric intake
Yassin et al, 2021	Ramadan: fasting from dawn to sunset	7	1 mo		N/A
PROTOCOL AND TRIAL REGISTRIES					
Christensen et al, 2022	16/8 TRE with the following variations: (1) adjustment of eating window length from 8 h up to 10 h; (2) self-selected eating window start time as long as end time is >3 h before bedtime; (3) follow TRE as many days as possible, but if time off is required, aim to limit it to 1–2 d off following at least 5 successive days of TRE each week.	5–7	Duration of chemotherapy (12–18 wks)		Standard of care, including a single group-based nutrition class, and provision of a copy of Canada’s Food Guide. The control group will maintain their usual dietary habits (timing, amount, and type) outside of any recommendations given in the nutrition class for the duration of the intervention period.
NCT03523377	Overnight fast of ≥14 h	7^a^	Daily × 6 mo		Eat a heart-healthy diet and exercise for at least 30 min for 5 d per wk, which is the usual counseling in our clinics
NCT04288336	16/8 TRE	7	1 y		N/A
NCT04560439	Modified Diabetes Prevention Program (DPP)-based lifestyle modification intervention that utilizes IF (no definition of IF)	7^a^	16 sessions over 6 mo		N/A
NCT04626843	16/8 TRE: fasting for 16 h of the day and eating with an 8-h feeding window	7^a^	3 mo		N/A
NCT04691999	TRE of ∼16–18 h fasting, 4 times per week	4	6 mo		N/A
NCT04708860	TRE with 13 h nocturnal fasting; no consumption of any calorie-containing food/drinks after 8 pm, goal of fasting at least 6 d per wk	6	12 wks		N/A
NCT04783467	TRE of 8–10 h eating window of prepared frozen lunch and dinner meals. The meal plans will be individualized to meet weight maintenance energy requirements.	7^a^	6 wks		For 4 wks, women will receive frozen lunch and dinner meals, and a standardized breakfast and snacks menu. The meal plans will be individualized to meet weight maintenance energy requirements. No restrictions on eating times.
NCT05023967	TRE of ≥16 nocturnal hours every night and use the continuous glucose monitoring system for 4–6 wks	7	4–6 wks		Patients continue their usual dietary pattern and use the continuous glucose monitoring system for 4–6 wks (until surgery).
NCT05083416	TRE in any 10-h eating period that falls between 6 pm and 6 pm It will be recommended that participants follow study diet guidelines, eat to satiety, and do not count calories. Participants will be allowed to have water, beverages (<4 kcal) during the fasting period.	7^a^	3 mo		A traditional eating pattern with no time restrictions
NCT05259410	16/8 TRE: a Mediterranean-style diet or usual diet will be eaten within the same self-selected 8-h eating window daily, with water fasting the remaining hours of the day	7^a^	12 wks		Standard care: eat enough calories and protein to maintain weight and lean mass
NCT05312255	16/8 TRE	7^a^	1 mo		Exercise: patients undergo strength training twice weekly for 6 mo, wear a FitBit, and increase physical activity over 6 mo
NCT05327608	TRE of 14 h fasting	7^a^	∼4 mo		N/A
NCT05722288	TRE Monday through Friday only (no eating/fasting windows provided)	5	4 wks		Nutritional counseling

a Number of days not explicitly stated but 7 days per week implied. *Abbreviations*: mo, month; N/A, not applicable; TRE, time-restricted eating.

Five articles utilized a single-arm pre/post design, with each participant serving as their own baseline control.^47–51^ Palomar-Cross and colleagues (2021) compared those who fasted for 11 or more hours overnight against those who fasted for less than 11 nocturnal hours. Similarly, Marnic et al (2016) compared a cohort who fasted for greater than 13 hours nocturnally against those who did not. Vega et al (2022) was the only study who compared against a different diet—caloric restriction. Participants in the comparator arm limited their intake to reduce calories to “25% of basal caloric intake.”

Most studies (*n* = 13) did not specify the number of fasting days per week; while 4 studies used daily fasting, 6 studies allowed for 1–3 non-fasting days per week (Table 3). Eight regimens limited eating windows to certain hours on the 24-hour clock, often aligned with participants’ sleep schedules (eg, last intake greater than 3 hours before bedtime).^51^ The duration of the fasts ranged from 2 to 12 weeks.

Of the trial protocols and registries, 75% (*n* = 9) applied 16/8 TRE, where an individual eats during an 8-hour window then fasts for 16 hours, with 3 of those protocols allowing a 10-hour eating window if participants found 8-hours too challenging. NCT05083416^52^ and NCT03523377^53^ both prescribed a 10-hour eating window (14 hour fast), while NCT04708860^54^ specified a 9-hour eating window (13 hour overnight fast). Two clinical trial registries did not specify the duration of the eating window.^55^^,^^56^ Only 2 clinical trial registries limited eating to specific hours of the 24-hour clock. The fasting duration ranged from 4 weeks to 1 year. Of the 10 completed studies, 3 (30%) were during Ramadan fasting,^44–46^ 3 (30%) applied 16/8 TRE,^47^^,^^48^^,^^57^ 2 (20%) applied a 13-hour nocturnal fast,^50^^,^^58^ and the remaining articles applied a 14-hour nocturnal fast,^49^ or a nocturnal fast of 11 or more hours.^59^ Regardless of the TRE regimen, there were high rates of adherence, ranging from 70% to 98%, with some participants adhering to the TRE 100% of the time (see Table 3).^47^^,^^48^

### Risk of bias

Of the 20 entries assessed, 45% (*n* = 9) were assessed as having a low risk of bias or being of “good” quality; 20% (*n* = 4) were assessed as having “some concerns” or were rated as having a “moderate” risk of bias; 20% (*n* = 4) were assessed as being of high concern; 15% (*n* = 3) were rated as having “very high,” “critical,” or “serious” risk of bias, based on the RoB tool predetermined criteria and definitions (Table 4^39–42^). See the online Supplementary material for the robvis visualizations. It should be noted, however, that the trial registries could not be comprehensively assessed, due to insufficient information, so the final rating may not fully reflect the study design in its entirety.

**Table 4.** Risk-of-Bias (RoB) Assessment Ratings (*n* = 20) (Discontinued Trial Registries [*n* = 4] Not Assessed)

Study ID	Tool	**Tool scale** ^39–42^	Rating
Alshammari et al, 2022	ROBINS-E	Low RoB except for concerns about uncontrolled confounding/some concerns/high RoB/very high RoB	High RoB
Badar et al, 2014	RoB2.0	Low RoB, some concerns, high RoB	High RoB
Christensen et al, 2022	RoB2.0	Low RoB, some concerns, high RoB	Some concerns
Kirkham et al, 2021	NIH	Poor/fair/good quality	Good quality
Kirkham et al, 2022	NIH	Poor/fair/good quality	Good quality
Kleckner et al, 2022	NIH	Poor/fair/good quality	Good quality
Marinac et al, 2016	ROBINS-E	Low RoB except for concerns about uncontrolled confounding/some concerns/high RoB/very high RoB	Very high RoB
O’Donnell et al, 2022	NIH	Poor/fair/good quality	Good quality
Palomar-Cros et al, 2021	ROBINS-E	Low RoB except for concerns about uncontrolled confounding/some concerns/high RoB/very high RoB	Low RoB
Vega et al, 2022	ROBINS-I	Low RoB/moderate RoB/serious RoB/critical RoB/no information	Critical RoB
Yassin et al, 2021	ROBINS-E	Low RoB except for concerns about uncontrolled confounding/some concerns/high RoB/very high RoB	High RoB
NCT03523377	RoB2.0	Low RoB, some concerns, high RoB	Low RoB
NCT04626843	NIH	Poor/fair/good quality	Good quality
NCT04708860	NIH	Poor/fair/good quality	Good quality
NCT04783467	RoB2.0	Low RoB/some concerns/high RoB	High RoB
NCT05023967	RoB2.0	Low RoB/some concerns/high RoB	Low RoB
NCT05083416	ROBINS-I	Low RoB/moderate RoB/serious RoB/critical RoB/no information	Serious RoB
NCT05259410	RoB2.0	Low RoB/some concerns/high RoB	Some concerns
NCT05312255	ROBINS-I	Low RoB/moderate RoB/serious RoB/critical RoB/no information	Moderate RoB
NCT05722288	RoB2.0	Low RoB/some concerns/high RoB	Some concerns

### Outcome assessments

There was heterogeneity in the end points and clinical outcomes assessed by the articles, protocols, and trial registries (Appendix SE).

### Clinical cancer markers

Only 4 of the 10 completed studies directly assessed a marker of cancer status as an end point. Alshammari and colleagues (2022) prospectively measured changes in carcinoembryonic antigen (CEA) and lactate dehydrogenase (LDH) in 37 patients with colorectal cancer who observed Ramadan, comparing results between those who fasted for 20 or more days against the results for those who fasted less than 20 days. CEA declined by −34.10 ng/mL (−40.9%) in the longer fasting group, compared with −1.93 ng/mL (−12.4%) in the shorter fasting group; however, the between-group difference was not significant (*P* = .24). LDH levels were comparable in the group that fasted for more than 20 days (−47.05 mmol/L [−15.7%]), compared with the group that fasted for less than 20 days (−50.29 mmol/L [−21.8%], *P* = .23).

The other 3 studies (2 cohort studies and 1 case–control study) that evaluated cancer outcomes were retrospective. Palomar-Cros et al (2021) evaluated data from 607 prostate cancer cases and 848 population controls from 7 different regions across Spain, comparing prostate cancer risk and aggressiveness, defined by the Gleason score, between those who fasted for greater or less than 11 hours at night. Fasting for greater than 11 hours per night was linked to lower odds of developing prostate cancer (adjusted model, OR = 0.77, 95% CI 0.54, 1.07) after adjusting for confounders. The impact of breakfast timing was also explored. Though not significant, individuals who consumed breakfast after 8:30 Am had higher odds of developing prostate cancer compared with those who consumed breakfast before 8:30 Am (OR = 1.30, 95% CI 0.92, 1.85). No significant differences in associations were found between fasting duration and low versus high Gleason score.

Marinac et al (2016) investigated whether the duration of nightly fasting predicted breast recurrence and mortality in 2413 women with early-stage, invasive breast cancer in a retrospective cohort study. The results demonstrated fasting duration to be associated with risk of breast cancer recurrence, but not breast cancer-specific or all-cause mortality. Specifically, women who fasted for less than 13 hours per night were at increased risk of breast cancer recurrence compared with women who fasted for 13 or more hours per night (hazard ratio, 1.36; 95% CI, 0.95 CI, 0.95–1.56). The duration of nightly fasting did not increase the risk of breast-cancer-specific mortality (hazard ratio, 1.21; 95% CI, 0.91–1.60) or all-cause mortality (hazard ratio, 1.22; 95% CI, 0.95–1.56).

Lastly, Yassin et al (2021) evaluated the effect of Ramadan fasting among chronic myelogenous leukemia (CML) patients receiving tyrosine kinase inhibitors (TKIs) by evaluating their clinical course, hematological parameters, and BCR-ABL1 levels, and found that Ramadan fasting had no effect on CML control. While white blood cells, neutrophils, basophils, and BCR-ABL were reduced after fasting compared with before and during, these differences were statistically insignificant (*P* > .05). The remaining hematological parameters, including mean values for platelets, hemoglobin, basophils, and eosinophils, maintained similar values before, during, and after Ramadan fasting. Patients achieved major molecular response in terms of the abnormal gene product in CML (BCR-ABL) before Ramadan and maintained it during and after Ramadan.

Among the published protocol and registries, 4 are measuring cancer markers, including cumulative double-strand deoxyribonucleic acid (dsDNA) damage of normal tissue and accumulated γH2ax foci, a marker of DNA damage^56^; change of proliferation genes and proteins, M30 antigen levels (a marker of residual tumor) and phosphorylated S6^60^; changes in lymphocyte count^61^; and changes in prostate-specific antigen (PSA) kinetics and/or doubling time.^62^

### Adiposity and glucoregulation

Metabolic factors, such as adipose tissue and glucose metabolism, that are demonstrated to be linked to cancer risk and/or aggressiveness was, the most heavily researched outcome, representing 32% of all end points. BMI and adiposity were the most common outcome measurements in this category. O’Donnell and colleagues (2022), who studied 40 patients with stage I–III invasive breast cancer, found a 13-hour overnight fast for 12 weeks resulted in statistically significant improvements in BMI, with a median within-participant decrease in BMI relative to baseline measurements of 0.38 kg/m^2^ (*P* = .007). In contrast, Marinac et al (2016) retrospectively assessed changes in BMI in 2413 patients with early stage I–III invasive breast cancer, who also undertook a nocturnal fast of 13 or more nightly hours, but found fasting duration was not associated with a change in BMI. However, they did find that each additional episode of intake (ie, eating episode) per day was associated with a significantly lower BMI, and that eating after 8 pm was associated with a significantly higher BMI. In 22 breast cancer survivors, Kirkham et al (2022) found 8 weeks of 16/8 TRE to have no significant effect on mean BMI (−0.2 ± 0.7 kg/m^2^; *P* = .10).^48^

Vega and colleagues (2022) and Kirkham and colleagues (2021 and 2022) assessed changes in waist circumference and visceral adipose tissues. Vega et al (2022) found a mean reduction in waist circumference of 4.9 cm^57^ among the 14 participants with overweight or obesity and either breast or prostate cancer who followed either 16/8 TRE or caloric restriction of 25% for 12 weeks. These results are reported as an absolute change only, so inference cannot be made about relative change, statistical significance, or difference between interventional arms. Similarly, Kirkham et al (2021) found that following 16/8 TRE on weekdays for 8 weeks resulted in significant decreases in mean magnetic resonance imaging–derived visceral adipose tissue (−5% ± 7%; *P* = .009), median bioelectric-impedance–derived whole-body fat mass (−0.9 kg; interquartile range [IQR]: −1.5 to 0.1 kg; *P* = .046), and median body mass (−1.0 kg; IQR: −2.3 to 0.2 kg; *P* = .025) across 22 breast cancer survivors.^48^ Despite these changes in adiposity, no significant, corresponding changes were observed in total cholesterol, high-density lipoprotein, or systolic blood pressure. However, in the same patient population, the former study of Kirkham and colleagues (2021) found a decrease of triglycerides by 17% (*P* = 0.008) across 15 patients.^47^

In terms of glucose regulation, Kirkham et al (2021) observed no changes in hemoglobin A1c (HbA1c), although their intervention was only 8 weeks long. Conversely, Vega et al (2022) found mean reductions in serum glucose, triglycerides, and insulin of 1.3 mg/dL, 24.2 mg/dL, and 2.1 uU/mL over 12 weeks. Marinac et al (2016) found that each 2-hour increase in nightly fasting duration was significantly associated with a 0.37-mmol/mol lower HbA1c level (β = −.37; 95% CI, .72 to .01, *P* = .04).

Kirkham et al (2021) also found that, among those at intermediate cardiovascular disease (CVD) risk (defined as a Framingham CVD risk score of 10% to 19%), 8 weeks of TRE decreased the absolute CVD risk by a median of 2% and heart age by 6 years.^47^ This amounted to reclassification to “low risk” in 45% of participants. Among the included trial registries, metabolic syndrome severity is a planned outcome of 1 trial.^51^

### Circulating biomarkers

Complete blood counts and inflammation were monitored in 3 studies. The most thorough biomarker assessment was by O’Donnell and colleagues (2022), who assessed an expanded lipid profile, leptin, adiponectin, HbA1c, C-reactive protein (CRP), interleukin-6 (IL-6), and tumor necrosis factor alpha (TNF-α). They found no significant changes in blood biomarkers by the end of the study, but did observe nonsignificant decreases in CRP, IL-6, and TNF-α, with mean within-person differences of −0.10 mg/L, −0.30 pg/mL, and −0.05 pg/mL, respectively. Alshammari et al (2022) had similar findings. No statistically significant changes were found in hemoglobin (Hgb), white blood cell count (WBC), platelets, estimated glomerular filtration rate (eGFR), alkaline phosphatase, or lactate dehydrogenase, in their 37 participants who followed Ramadan fasting. In their nonrandomized crossover study, Badar and colleagues (2014) found blood biomarkers related to safety were maintained within the normal range when fasting, compared with nonfasting (WBC −0.30 × 10^9^/L [95% CI −3.87, 3.25], absolute neutrophil count [ANC] −0.14 × 10^9^/L [95% CI −2.89, 2.57]).

### Quality of life

QOL measures were assessed in 4 articles (40%) but are planned for assessment in 8 (57%) of the trial protocols and registries. Both O’Donnell et al (2022) and Kleckner et al (2022) monitored fatigue with the Functional Assessment of Chronic Illness Therapy–Fatigue (FACIT-F) tool,^63^^,^^64^ and both found significantly decreased levels of fatigue at the end of the TRE intervention, compared with the prefasting baseline. Kleckner et al (2022) found TRE for 14 consecutive days resulted in improvements on the FACIT-F fatigue subscale of 5.3 ± 8.1 points (*P* < .001), representing a clinically meaningful increase. O’Donnell et al (2022) noted that improvements in fatigue scores were maintained at the end of the 12-week trial (*P* = .0105).

Kleckner and colleagues (2022) also found significant pre/post improvements in physical well-being (1.4 ± 2.9 points), functional well-being (1.7 ± 3.3 points), and QOL as measured from the Functional Assessment of Cancer Therapy–General (FACT-G) subscale (4.0 ± 7.0 points, *P* < .01). Vega et al (2022) also monitored QOL in both of their interventional arms; however, findings pertaining to QOL were not included in the results of their primary report. Lastly, Marnic et al (2016) found that each 2-hour increase in nightly fasting duration was associated with more hours of sleep per night.

Of the published protocol and trial registries, assessment of psychological well-being, such as stress and anxiety, is mentioned in 2 reports.^60^^,^^65^ Otherwise QOL is planned to be measured globally with the European Organization for Research and Treatment of Cancer tools being utilized in 5 of the 8 trials assessing QOL.^54^^,^^56^^,^^61^^,^^65–67^ Other QOL markers include changes in functional status,^68^ treatment side effects,^51^^,^^67^ and blood pressure.^66^

### Gut microbiome and metabolomics

None of the published articles studied the gut microbiome or metabolomics; however, 3 of the trial registries plan to include them. The trial by the Roswell Park Cancer Institute will compare changes in the gut microbiome, from fecal samples, comparing those on the TRE arm against those on the exercise arm,^65^ while the studies by BC Cancer^53^ and the H. Lee Moffitt Cancer Center and Research Institute^61^ will study changes in the microbiome and gut metabolites before and after TRE.

## DISCUSSION

This systematic review of the literature summarizes the evidence on the effects of TRE in people with cancer, compared with ad libitum eating or an alternative diet, such as caloric restriction. Of the 24 studies, only 10 have been completed, with the remainder being clinical protocols and trial registries. Of the completed studies, there was heterogeneity among the study populations, TRE regimens, and outcomes measures, so conclusions on the effects of TRE on cancer-related outcomes cannot yet be drawn. Despite this, these preliminary findings suggest that TRE is feasible in this population and may have clinical health benefits for people with cancer, while also improving QOL. The study designs and end points of the published protocol and trial registries consider clinical outcomes, and 3 studies also include microbiome or metabolomics assessments.

### Optimal duration of fast

To reduce heterogeneity in the IF regimens, our study limited the criteria to TRE, but there was still inconsistency in how TRE was applied. The interventions varied in the length of the fasting/eating window, the duration of the intervention, and the timing on the 24-hour clock (eg, intake must stop by 8 pm). While the popular 16/8 TRE was more frequently applied,^47^^,^^48^^,^^51^^,^^57^^,^^60–62^^,^^65–68^ the eating window ranged from 8 to 11 hours. Physiological response to fasting suggests that some individuals will deplete their glycogen stores within 12 hours, necessitating the “metabolic switch” from carbohydrates to fat as energy; however, for many individuals a longer fast may be required, hence the rationale for fasting 16 hours. None of the studies checked for ketone bodies, an indicator of ketosis in this fasting state, so no conclusions can be drawn comparing the efficacy of an 8-hour fast against that of an 11-hour fast. This is a gap that future studies should consider addressing.

The 2 large cohort studies, however, offer some insight into the different physiological responses based on the duration of the fasting window. The 2 cohort studies compared fasting for more than 11 hours with fasting for less than 11 hours,^59^ and fasting for more than 13 hours with fasting for less than 13 hours^58^ and still found significantly lower odds of prostate cancer and lower risk of breast cancer recurrence, respectively. This suggests that a fast as long as 16 hours may not be necessary for cancer prevention and control. However, given the observational nature of the studies, additional, randomized controlled studies are needed to elucidate the ideal fasting window for optimal health outcomes that is still acceptable to patients.

### Cancer outcomes

While conclusions on cancer outcomes cannot be drawn from the limited evidence presented in this review, all 4 articles that measured a direct cancer marker or other indicator of cancer control suggested a beneficial effect of TRE on cancer. Fasting for greater than 13 hours was found to be associated with reduced risk of breast cancer recurrence by 36%.^58^ While it is plausible that longer nightly fasting is also associated with eating patterns and behaviors that align with the American Institute for Cancer Research guidelines for primary and tertiary cancer prevention,^2^ such as less alcohol intake, less consumption of high-fat and high-sodium foods, and greater physical activity, their models adjusted for these factors. When accounting for other aspects of diet, including the number of eating episodes per day, eating after 8 pm, kilocalorie intake, total fat and carbohydrate intake, and dietary index scores, along with comorbidities, tamoxifen use, and menopausal status, the multivariate adjusted models remained statistically significant, suggesting the protective association is indeed due to TRE.^58^ This protective association is consistent with the preliminary evidence that exists for other forms of IF on breast cancer. For example, Caffa et al (2021) found that periodic fasting (weekly cycles of 48-hour water-only fasting) enhanced tamoxifen and fulvestrant anticancer activity in mouse xenografts of several HR+/HER2− breast cancer cell lines.^69^

Palomar-Cros et al (2021) also adjusted for confounders, considering dietary factors such as adherence to a healthy lifestyle through the World Cancer Research Fund/American Institute for Cancer Research score, timing of eating, and number of daily intake episodes.^56^ After adjusting for confounders, the model showed that a more extended nightly fast was linked to lower odds of prostate cancer (adjusted model, OR = 0.77, 95% CI 0.54, 1.0), yet not prostate cancer aggressiveness. Additional controlled, clinical studies are needed on TRE and prostate cancer outcomes to verify these findings. Future studies would benefit from a longitudinal design with measurement of direct markers of cancer progression through imaging or laboratory assessment (eg, carcinoembryonic antigen [CEA], cancer antigen 125 [CA-125], alpha-fetoprotein [AFP], PSA, circulating transfer DNA). Collection of confounding variables, as in Palomar-Cross et al and Marinac et al, would help to further elucidate the potential benefits of TRE on cancer independent of dietary behavior changes, such as total calorie intake and food choices.

### Secondary risk factors

It appears TRE may have a favorable impact on BMI, reducing waist circumference and visceral adipose tissue^50^; however, the data also suggests that the timing of eating, known as chrononutrition, and number of meals per day also influence measures of body composition.^45^ TRE in other populations, such as overweight or obese adults,^70^^,^^71^ healthy adults,^72^^,^^73^ and athletes,^74^^,^^75^ demonstrate a similar trend of TRE reducing overall body weight, yet maintaining lean body mass. A 2020 meta-analysis of TRE in people with metabolic disease found participants following TRE had significantly reduced body weight (mean difference −0.90; 95% CI: −1.71 to −0.10) and fat mass (mean difference −1.58, 95% CI: −2.64 to −0.51), while preserving fat-free mass (mean difference −0.24; 95% CI: −1.15 to 0.67).^76^ In a subgroup analysis of 5 studies on TRE, weight loss was found to be greatest in those with metabolic abnormalities (ie, overweight or obesity, pre-diabetes, metabolic syndrome).^13^ This can hold implications for oncology, as metabolic abnormalities are both a risk factor and a result of some cancers and/or cancer treatments, such as the development of metabolic syndrome from treatment with androgen deprivation therapy for prostate cancer.

Only half (*n* = 2 of 4) of the articles that assessed changes in glucoregulation found a significant decrease in HbA1c^58^ or blood glucose and insulin.^57^ It must be noted, however, that red blood cells turn over approximately every 3 months, so a 3-month time frame may be required in order to observe a difference in glycated hemoglobin. This time requirement likely accounts for the lack of significant changes in HbA1c noted by both Kirkham et al (2021) during their 8-week TRE intervention and by Alshammari et al (2022) during 30 days of Ramadan. Future studies should consider implementing TRE for a minimum of 3 months to allow sufficient time to observe any changes in glycated hemoglobin, or consider using a different marker to assess glucoregulation, such as fasting blood glucose, HOMA-IR, or 1,5-anhydroglucitol, which have a short assessment period.^77^

While statistically significant reductions in inflammation were not found following TRE in the 2 studies that monitored inflammation, Marinac et al (2016) did note that eating after 8 pm was associated with significantly higher CRP concentrations. This supports the theory developed in mechanistic literature^78^ that improvements in inflammation are due to alignment of eating with the circadian rhythm, as many metabolic and hormonal rhythms peak in the mornings.^34^^,^^79^ In contrast, studies on Ramadan fasting, where intake occurs between dusk and dawn, in opposition of the circadian rhythm, are of mixed results. For example, Aksungar et al (2007) reported a statistically significant decrease in IL-6, CRP, and homocysteine following Ramadan in 40 health adults of normal weight compared with their basal levels,^80^ while a similar observational study was performed in 30 healthy men yet no significant differences were found for IL-6 or high-sensitivity CRP.^81^ A 2023 review of 5 human studies on the effect of IF on circulating inflammatory markers in obesity concluded that TRE, with various eating window durations, had no effect on CRP, TNF-α, or IL-6 when accompanied with 1% to 5% weight loss. In contrast, an exploratory study of 17–19 hour daily fasts for 30 days in 25 healthy, young males induced the expression of the inflammasome genes NLRP3 and IL-1ß within 2 weeks of fasting. Expression returned to basal levels after 1 week of ending the fast.^82^ Ultimately, more research is needed to better understand the possible role of TRE on inflammation, through using a comprehensive and sensitive set of biomarkers to draw a clearer picture of the inflammatory response, which is complex in nature.^83^

### Microbiome and metabolomics

Although this systematic review did not uncover any results related to the microbiome or metabolomics, the microbiome is an end point in 2 of the clinical trial registries.^53^^,^^61^ The published literature demonstrates the metabolome to be a responsive system that gradually changes upon duration and level of nutrient deprivation.^8^ In their systematic review of studies looking at the effects of IF on the microbiota, Mousavi et al (2022) found there was a significant shift in the microbiota flora resulting from IF (*n* = 28).^22^ Furthermore, the composition of the microbiome heavily influences bile acid metabolism, which can result in products such as deoxycholic acid (DCA), lithocholic acid, and urodeoxycholic acid (UDCA), which have direct implications for carcinogenesis of various cancer types.^24^^,^^84^^,^^85^ For example, UDCA is demonstrated to be a cancer inhibitor,^86–89^ while DCA is a cancer activator.^90–92^ These metabolites were shown to be impacted in people with chronic lymphocytic leukemia and small lymphocytic lymphoma in a feasibility trial of TRE, where 15 participants adhered to 16/8 TRE for either 3 or 6 months. Significant changes in the secondary bile acids UDCA and DCA, and a branched-chain fatty acid, isovaleric acid, were discovered.^93^

Taxonomic differences have also been shown to influence the effectiveness of immunotherapies using checkpoint inhibitors. Nomura et al (2020) found that short-chain fatty acids, including fecal acetic acid, propionic acid, butyric acid, valeric acid, and plasma isovaleric acid were associated with longer progression-free survival after treatment with programmed cell death 1 inhibitors (PD-1is).^26^ This indicates a clinical potential for using IF as a modifiable factor in the gut microbiome, relevant not only to tumorigenesis but also to the effectiveness of immunotherapies. The recent inclusion of the microbiome as an end point in clinical trials may help to clarify the potential mediating role of the microbiome.

### Clinical implications

Based on the results of this review, it is premature to recommend the use of TRE for people with cancer. More studies are needed to elucidate the ideal fasting regimen that balances clinical and metabolic benefits with safety and the patient’s perspective on acceptability. While adherence was reported, none of the studies included qualitative assessment of the patient’s experience with TRE.

Furthermore, people with cancer often struggle with adequate nutritional intake, which can lead to or exacerbate malnutrition and sarcopenia.^94^ Patients defined as being at risk for developing malnutrition should receive an individualized, comprehensive nutritional assessment and treatment plan.^95^ While a high-protein and high-calorie diet can be followed on TRE, the limited hours of intake may prevent maximal caloric intake. Future studies should consider including dietary assessment and body composition analysis to determine whether adequate protein and calories can be consumed and whether lean body mass can be maintained on TRE in people with cancer.

With a lack of evidence to define the suitability of TRE for specific cancer populations, caution should be applied with patients following TRE, closing monitoring for physical or biochemical signs of nutritional deficiencies. Due to the nutritional challenges that frequently accompany advanced stages of cancer and high-risk treatment protocols, TRE will not be appropriate for all patients. TRE is likely most appropriate for those with consistently adequate nutritional intake and a stable weight.

### Future directions

With the exception of the 2 cohort studies, the sample size of the completed studies was small (*n* < 50). However, the trial registries demonstrate growing momentum and progress in the field: 3 phase II trials, 1 phase I trial, and 1 early phase I trial, with target sample sizes ranging from 15 to 150 participants. Furthermore, the protocol and clinical trial registries include 5 RCTs, which will be the first RCTs on TRE in people with cancer. This advancement suggests the establishment of feasibility, while the advent of RCTs will help determine causality. Additionally, 2 of these trials are likely adequately powered, with 150 (NCT05312255) and 120 (NCT05023967) planned participants, to detect differences in outcomes. This will make a momentous contribution, as causality has yet to be explored.

While a strength of this literature review is the narrowing of the broad conceptualization of IF to strictly TRE interventions, the review process is limited by the small number of studies. Currently, the field of TRE in cancer is in its infancy. The results to date demonstrate the hitherto lack of consistency in how TRE is applied, the tumor site it is tested on, the outcomes of interest, and how the effects of TRE are being measured. The sparseness and heterogeneity of the literature prevent comparison across studies.

To advance the field, researchers first need to work towards identifying the most effective TRE regimens that are feasible and acceptable for patients. This can be done by monitoring for ketone bodies to confirm that the “metabolic switch” occurs with the given fasting window and by including qualitative feedback from participants for insight into the acceptability of the fasting window. Standardization of TRE regimens and outcome measures are critical for study comparisons. Second, studies should focus on highly metabolically active tumor groups that are also at lower malnutrition risk, for safety reasons, such as triple-negative (ER/PR/HER2-negative) breast cancers, melanoma, and high-grade lymphomas. Such cancers generally are highly avid for fludeoxyglucose F18 (FDG), a radiolabeled glucose analog, indicating their high metabolism and/or dependence on glucose. It is within this group that dietary interventions targeting energy utilization may hold the greatest promise. Third, appropriate comparator groups are needed within the study designs. To date, only half of the completed studies (*n* = 5 of 10) and 57% (*n* = 8 of 14) of protocols and trial registries have a comparator group, which makes it challenging to compare efficacy of TRE relative to standard of care. Lastly, in order to confirm the theorized, underpinning biological mechanisms, future studies should consider incorporating biomarkers of cancer-related outcomes and measures of the gut microbiome and metabolomics.

## CONCLUSION

In conclusion, evidence identified in this systematic review suggests a potential benefit of TRE for people with cancer in terms of clinical cancer outcomes, cancer risk factors, and QOL, although the number of studies and numbers of patients within studies are limited. Additionally, TRE appears to be highly feasible and safe in the 10 studies completed thus far. No completed studies have reported changes to the microbiome or the resulting metabolites; however, this is a planned outcome measure in 2 clinical trial registries. More clinical trials that move beyond feasibility are needed to elucidate the effect of TRE in people living with cancer.

## Supplementary Material

nuae105_Supplementary_Data

## Data Availability

The protocol and data described in the manuscript will be made available upon request pending application and approval.
