# Supplementary material for: The Clinical Impact of Time-restricted Eating on Cancer: A Systematic Review
Source: Nutr Rev. 2024 Aug 30;83(7):e1660–76. doi: 10.1093/nutrit/nuae105 (PMC12166167; doi:10.1093/nutrit/nuae105)
Supplement: nuae105_Supplementary_Data [file nuae105_supplementary_data.zip › nuae105_Supplementary_Data/Nutrition Reviews_Supplemental Information_July 4 2024.docx]

**SUPPLEMENTAL INFORMATION**

**Table S1. Core concepts of search terms are based on three central concepts**

| **Intermittent Fasting** | **Cancer** | **Clinical Trial** |
| --- | --- | --- |
| Time restricted feeding | Oncology | Interventions |
| 16/8 Method | Neoplasm | Observational |
| Alternate feeding | Tumor | Pilot |
| Fasting |  | Feasibility |
| Ramadan |  | Prevention |
| Muslim |  | Treatment |

**Appendix S1. Database search strategies.**

**MEDLINE**

( TITLE-ABS-KEY ( cancer*  OR  neoplasm*  OR  oncolog* )  AND  TITLE-ABS-KEY ( fasting  OR  "intermittent fast*"  OR  "time restrict* feed*"  OR  ( diet*  AND  fast* )  OR  ramadan  OR  ( muslim*  W/2  fast* ) )  AND  TITLE-ABS-KEY ( human*  OR  patient* ) )

**Embase**

Database: Embase <1974 to 2023 January 11>
Search Strategy:
--------------------------------------------------------------------------------
1     exp Neoplasm/ (5290494)
2     (neoplasm* or cancer* or oncolog*).tw,kf. (3404079)
3     1 or 2 (5951672)
4     exp fasting/ (21619)
5     ("intermittent fast*" or "time restrict* feed*" or (diet and fast*) or Ramadan or (Muslim* adj3 fast*)).tw,kf. (39047)
6     4 or 5 (56435)
7     3 and 6 (3958)
8     limit 7 to "humans only (removes records about animals)" (3440)

***************************

**CINAHL**

| **#** | **Query** | **Limiters/Expanders** |
| --- | --- | --- |
| S7 | S3 AND S6 | Search modes - Boolean/Phrase |
| S6 | S4 OR S5 | Search modes - Boolean/Phrase |
| S5 | TI ( ( fasting or Ramadan or (Muslim* N3 fast*) ) ) OR AB ( ( fasting or Ramadan or (Muslim* N3 fast*) ) ) | Search modes - Boolean/Phrase |
| S4 | (MH "Fasting") OR (MH "Ramadan") | Search modes - Boolean/Phrase |
| S3 | S1 OR S2 | Search modes - Boolean/Phrase |
| S2 | AB ( cancer* or neoplasm* or oncolog* or tumo#r* or malignanc* ) OR TI ( cancer* or neoplasm* or oncolog* or tumo#r* or malignanc* ) | Search modes - Boolean/Phrase |
| S1 | (MH "Neoplasms+") OR (MH "Cancer Survivors") OR (MH "Cancer Patients") | Search modes - Boolean/Phrase |

**CENTRAL**

#SearchesResults1exp Neoplasms/897982(neoplasm* or cancer* or oncolog*).tw,kf.17706031 or 22092114fasting/34965("intermittent fast*" or "time restrict* feed*" or (diet and fast*) or Ramadan or (Muslim adj3 fast*)).tw,kf.855464 or 51141373 and 6630

**Web of Science**

cancer* or neoplasm* or oncolog* (Topic) and fasting or "intermittent fast*" or "time restrict* feed*" or (diet* and fast*) or Ramadan or (Muslim NEXT/3 fast*) (Topic)

**Scopus**

( ( ( ( ( TITLE ( cancer*  OR  neoplasm*  OR  oncolog* )  OR  ABS ( cancer*  OR  neoplasm*  OR  oncolog* ) ) )  AND  ( ( TITLE ( fasting  OR  "intermittent fast*"  OR  "time restrict* feed*"  OR  ( diet*  AND  fast* )  OR  ramadan  OR  ( muslim*  W/2  fast* ) )  OR  ABS ( fasting  OR  "intermittent fast*"  OR  "time restrict* feed*"  OR  ( diet*  AND  fast* )  OR  ramadan  OR  ( muslim*  W/2  fast* ) ) ) ) )  AND  ( ( TITLE ( ( human*  OR  patient*  OR  participant* ) )  OR  ABS ( ( human*  OR  patient*  OR  participant* ) ) ) ) )  AND NOT  ( ( TITLE ( "polycystic ovar* syndrome*"  OR  pcos )  OR  ABS ( "polycystic ovar* syndrome*"  OR  pcos ) ) ) )  AND NOT  ( ( TITLE ( diabet* )  OR  ABS ( diabet* ) ) )

**Appendix S2. Full reference list of included articles.**

Alshammari K., Alhaidal H., Alharbi R., Alrubaiaan A., Alyousif G., Alkaiyat M., & Abdel-Razaq W.S. (2022). The Impact of Fasting the Holy Month of Ramadan on Colorectal Cancer Patients and Two Tumor Biomarkers: A Tertiary-Care Hospital Experience. *MedRxiv*, *(Alshammari, Alyousif, Alkaiyat) King Abdulaziz Medical City, Ministry of National Guard Health Affairs, Riyadh, Saudi Arabia(Alhaidal, Abdel-Razaq) College of Pharmacy, King Saud bin Abdulaziz University for Health Sciences, Ministry of National Guard He*. https://doi.org/10.1101/2022.08.04.22278413

Badar, T. (2014). Safety and Feasability of Muslim Fasting While Receiving Chemotherapy. *IOSR Journal of Pharmacy (IOSRPHR)*, *4*(1), 15–20. https://doi.org/10.9790/3013-0401015-20

Champ, C. The effect of intermittent fasting on body composition in women with breast cancer (EFFECT-BC). ClinicalTrials.gov identifier: NCT04691999. Updated November 21, 2021. Accessed September 21, 2023. https://www.clinicaltrials.gov/study/NCT04691999

Chung, C. Effect of prolonged nightly fasting on immunotherapy outcomes of HNSCC – role of gut microbiome. ClinicalTrials.gov identifier: NCT05083416. Updated July 3, 2023. Accessed September 26, 2023. https://www.clinicaltrials.gov/study/NCT05083416

Christensen, R. A., Haykowksy, M. J., Nadler, M., Prado, C. M., Small, S. D., Rickard, J. N., Pituskin, E., Paterson, D. I., Mackey, J. R., Thompson, R. B., & Kirkham, A. A. (2022). Rationale and Design of IMPACT-women: A randomized controlled trial of the effect of time-restricted eating, healthy eating, and reduced sedentary behavior on metabolic health during chemotherapy for early-stage breast cancer. *The British Journal of Nutrition*, *az4, 0372547*, 1–20. https://doi.org/10.1017/S0007114522003816

Friedman, D. Overnight fasting after completion of therapy: The OnFACT study. ClinicalTrials.gov identifier: NCT03523377. Updated August 21, 2023. Accessed September 21, 2023. https://www.clinicaltrials.gov/study/NCT03523377

Gabel, K. Time restricted eating during chemotherapy for breast cancer. ClinicalTrials.gov identifier: NCT05259410. Updated April 13, 2023. Accessed September 26, 2023. https://www.clinicaltrials.gov/study/NCT05259410

Hillengass, J. Non-chemotherapeutic interventions for the improvement of quality of life and immune function in patients with multiple myeloma. ClinicalTrials.gov identifier: NCT05312255. Updated March 1, 2023. Accessed September 26, 2023. https://www.clinicaltrials.gov/study/NCT05312255

James, R. K. Daily, Long-term intermittent fasting for the prevention of PSA-recurrence in patients with localized prostate cancer after radical prostatectomy. ClinicalTrials.gov identifier: NCT04288336. Updated July 11, 2022. Accessed September 21, 2023. https://www.clinicaltrials.gov/study/NCT04288336

Kirkham, A. A., Ford, K. L., Topolnyski, J., Da Silva, B. R., Paterson, D. I., Prado, C. M., Joy, A. A., Boule, N. G., Pituskin, E., Haykowsky, M. J., & Thompson, R. B. (2022). Time-Restricted Eating to Reduce Cardiovascular Risk Among Older Breast Cancer Survivors: A Single-Arm Feasibility Study. *JACC. CardioOncology*, *4*(2), 276–278. https://doi.org/10.1016/j.jaccao.2022.03.002

Kirkham A.A., Topolnyski J., Haykowsky M.J., Paterson I., Prado C.M., Joy A.A.A., Boule N., Ford K.L., Andre P.B., Da Silva B.R., Pituskin E., & Thompson R. (2021). Weekday 16:8 time-restricted eating in breast cancer survivors: Feasibility, safety, and effects on cardiometabolic health. *Circulation*, *144*(SUPPL 1). https://doi.org/10.1161/circ.144.suppl-1.12484

Kleckner, A. S., Altman, B. J., Reschke, J. E., Kleckner, I. R., Culakova, E., Dunne, R. F., Mustian, K. M., & Peppone, L. J. (2022). Time-restricted Eating to Address Cancer-related Fatigue among Cancer Survivors: A Single-arm Pilot Study. *Journal of Integrative Oncology*, *11*(5).

Li, Y. R. Time-restricted eating versus nutritional conseling for the reduction of radiation or chemoradiation tx side effects in patients with prostate, cervical or rectal cancers. ClinicalTrials.gov identifier: NCT05722288. Updated August 24, 2023. Accessed September 26, 2023. https://www.clinicaltrials.gov/study/NCT05722288

Ligibel, J. Feasibility of fasting and exercise in patients with HR+ MBC. ClinicalTrials.gov identifier: NCT04708860. Updated November 29, 2022. Accessed September 21, 2023. https://www.clinicaltrials.gov/study/NCT04708860

Marinac, C. R., Nelson, S. H., Breen, C. I., Hartman, S. J., Natarajan, L., Pierce, J. P., Flatt, S. W., Sears, D. D., & Patterson, R. E. (2016). Prolonged Nightly Fasting and Breast Cancer Prognosis. *JAMA Oncology*, *2*(8), 1049–1055. https://doi.org/10.1001/jamaoncol.2016.0164

O’Donnell E., Shapiro Y., Comander A., Isakoff S., Moy B., Spring L., Wander S., Kuter I., Shin J., Specht M., Kournioti C., Hu B., Sullivan C., Winters L., Horick N., & Peppercorn J. (2022). Pilot study to assess prolonged overnight fasting in breast cancer survivors (longfast). *Breast Cancer Research and Treatment*, *193*(3), 579–587. https://doi.org/10.1007/s10549-022-06594-4

Palomar-Cros, A., Espinosa, A., Straif, K., Perez-Gomez, B., Papantoniou, K., Gomez-Acebo, I., Molina-Barcelo, A., Olmedo-Requena, R., Alguacil, J., Fernandez-Tardon, G., Casabonne, D., Aragones, N., Castano-Vinyals, G., Pollan, M., Romaguera, D., & Kogevinas, M. (2021). The Association of Nighttime Fasting Duration and Prostate Cancer Risk: Results from the Multicase-Control (MCC) Study in Spain. *Nutrients*, *13*(8). https://doi.org/10.3390/nu13082662

Playdon, M. Time restricted eating (TRE) among endometrial cancer patients (TREND). ClinicalTrials.gov identifier: NCT04783467. Updated November 7, 2022. Accessed September 21, 2023. https://www.clinicaltrials.gov/study/NCT04783467

Samoa, R. Diabetes prevention program (METFIT) in reducing insulin resistance in stage I-III breast cancer survivors. ClinicalTrials.gov identifier: NCT04560439. Updated June 9, 2022. Accessed September 21, 2023. https://www.clinicaltrials.gov/study/NCT04560439

Stringer, E. Intermittent fasting and CLL/SLL. ClinicalTrials.gov identifier: NCT04626843. Updated February 14, 2023. Accessed September 21, 2023. https://www.clinicaltrials.gov/study/NCT04626843

Thomas, P. Metformin and nightly fasting in women with early breast cancer. ClinicalTrials.gov identifier: NCT05023967. Updated June 23, 2023. Accessed September 21, 2023. https://www.clinicaltrials.gov/study/NCT05023967

Vega C. & Merino T. (2022). Caloric restriction or intermittent fasting in cancer patients receiving curative radiotherapy. *Radiotherapy and Oncology*, *170*(Supplement 1), S1028–S1029. https://doi.org/10.1016/S0167-8140%2822%2903178-4

Yassin, M. A., Ghasoub, R. S., Aldapt, M. B., Abdulla, M. A., Chandra, P., Shwaylia, H. M., Nashwan, A. J., Kassem, N. A., & Akiki, S. J. (2021). Effects of Intermittent Fasting on Response to Tyrosine Kinase Inhibitors (TKIs) in Patients With Chronic Myeloid Leukemia: An Outcome of European LeukemiaNet Project. *Cancer Control : Journal of the Moffitt Cancer Center*, *28*(dlg, 9438457), 10732748211009256. https://doi.org/10.1177/10732748211009256

NA. Intermittent fasting for the improvement of outcomes in patients with stage I-III breast cancer receiving chemotherapy before surgery. ClinicalTrials.gov identifier: NCT05327608. Updated January 26, 2023. Accessed September 26, 2023. https://www.clinicaltrials.gov/study/NCT05327608

**Figure S1. Robvis visualizations**


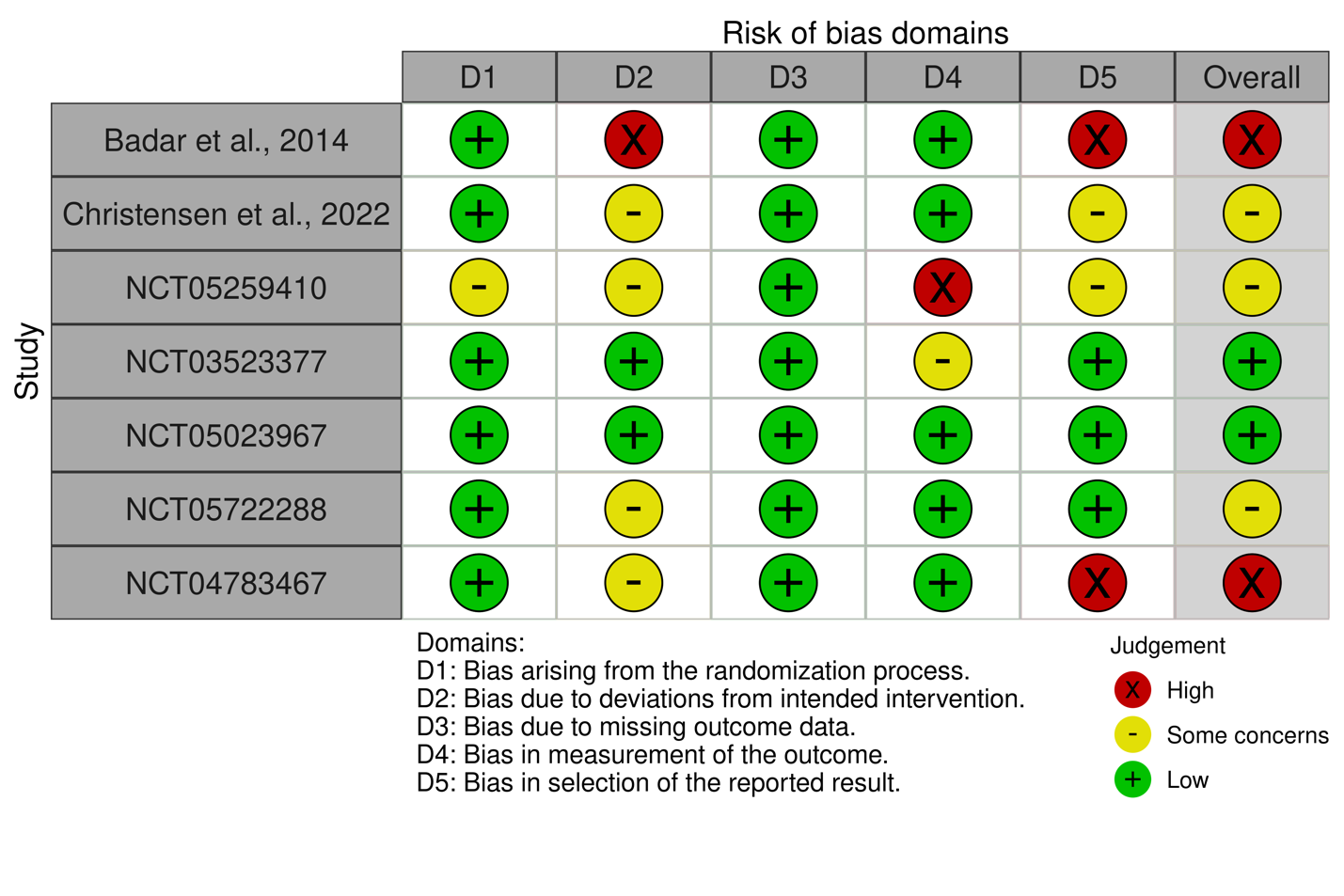


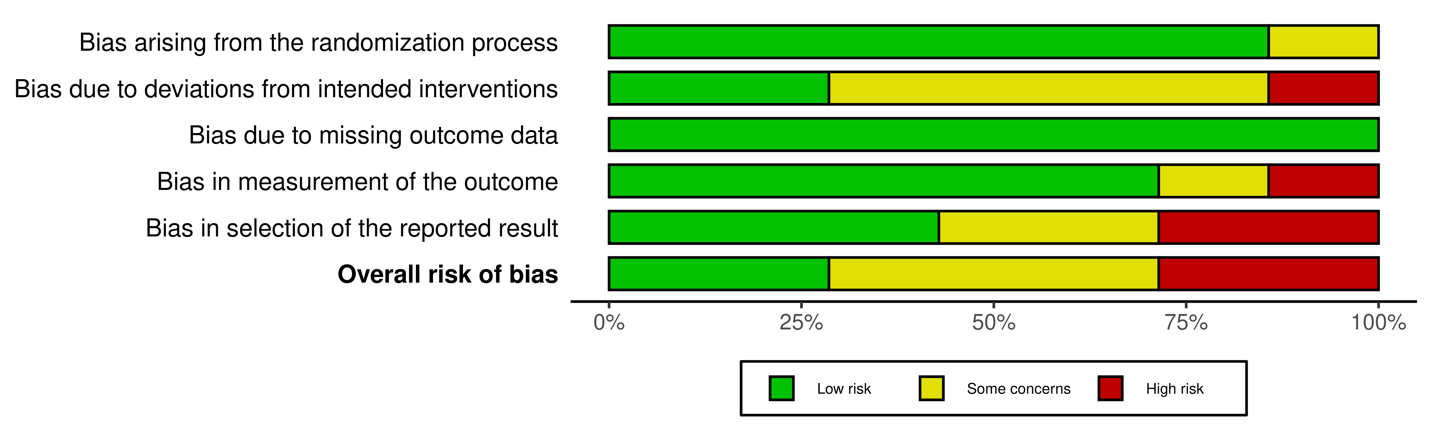


**
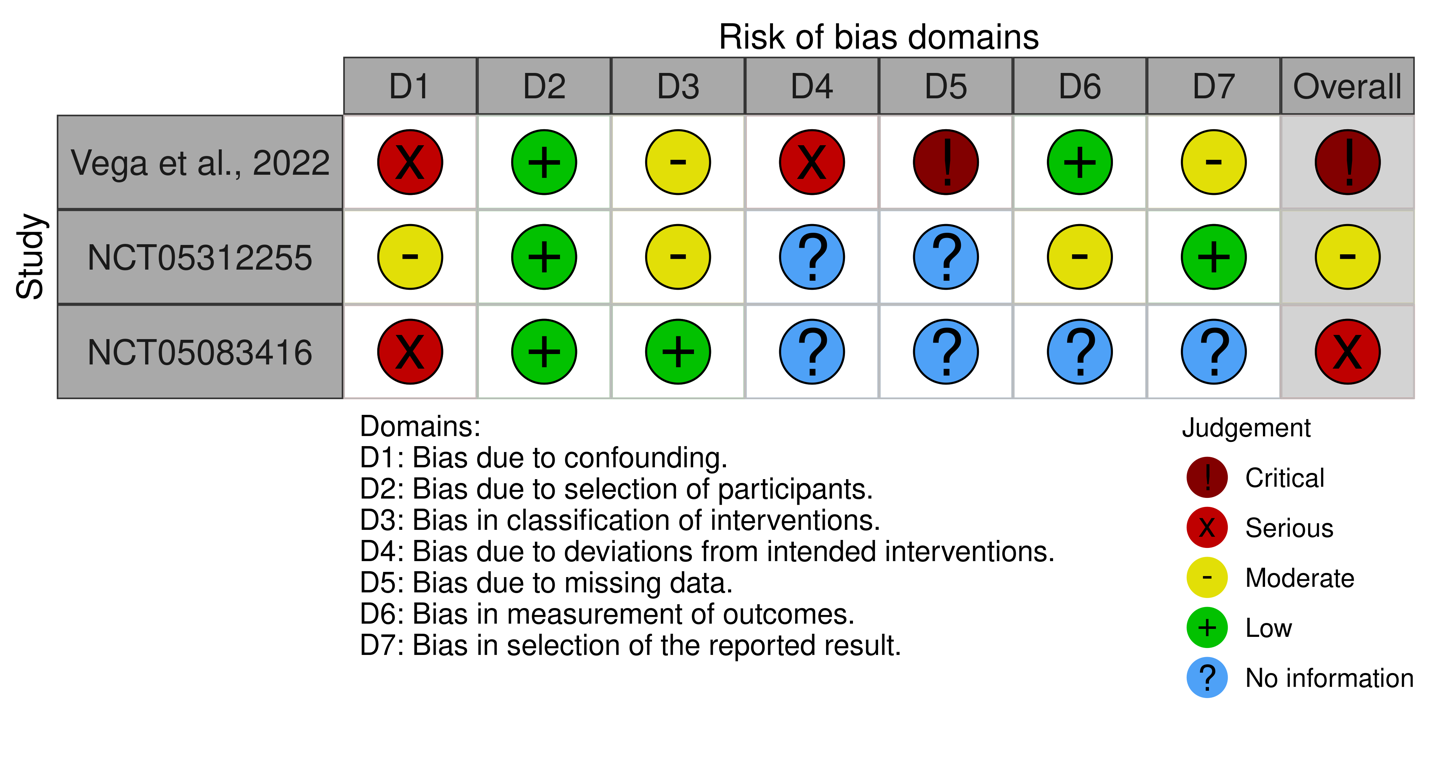

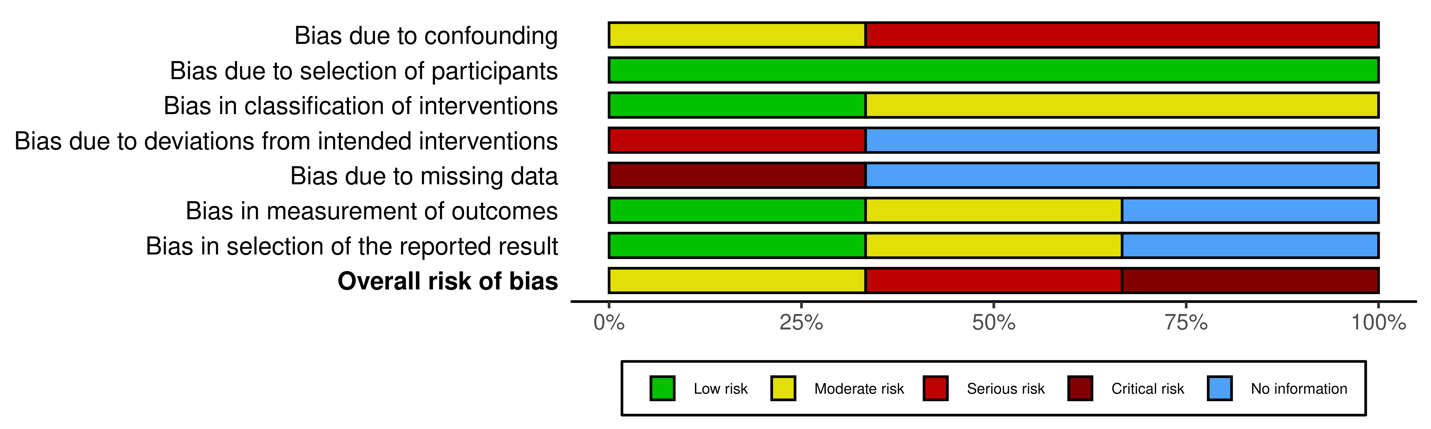
**


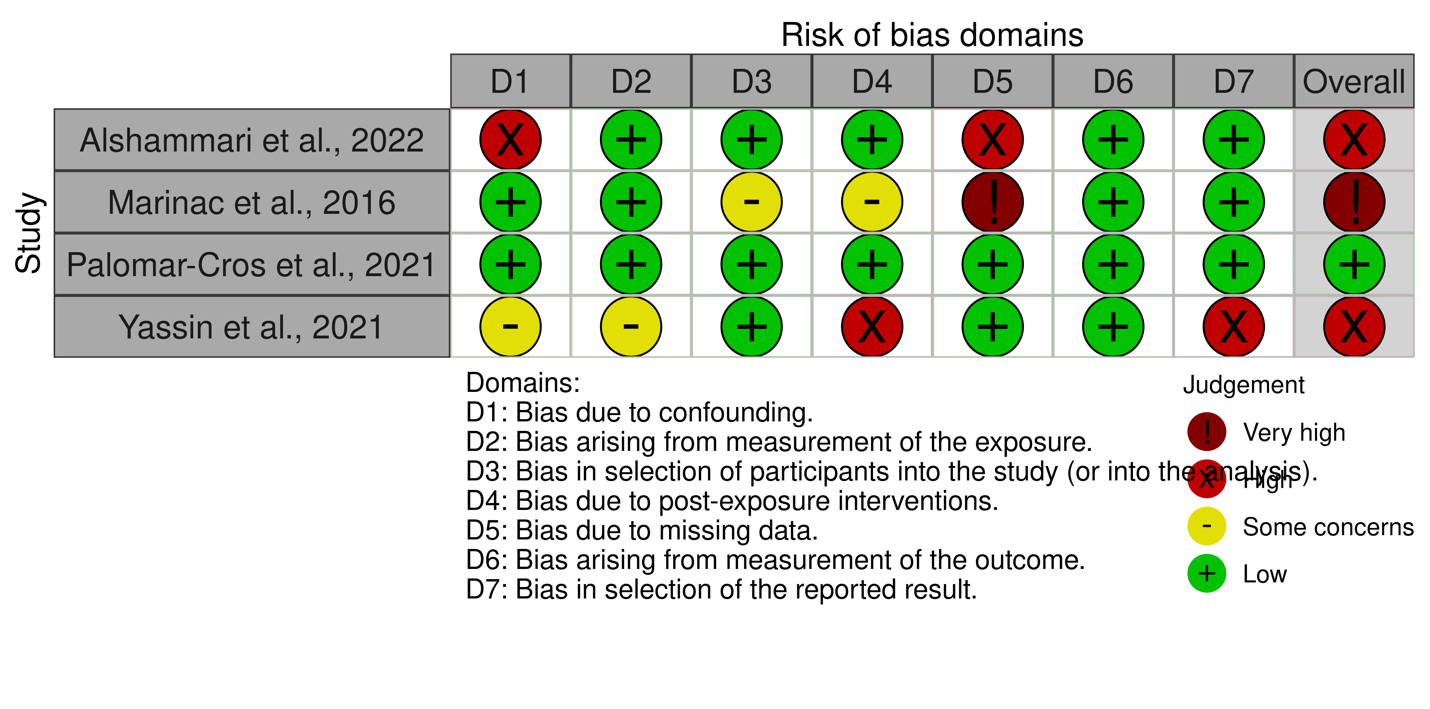


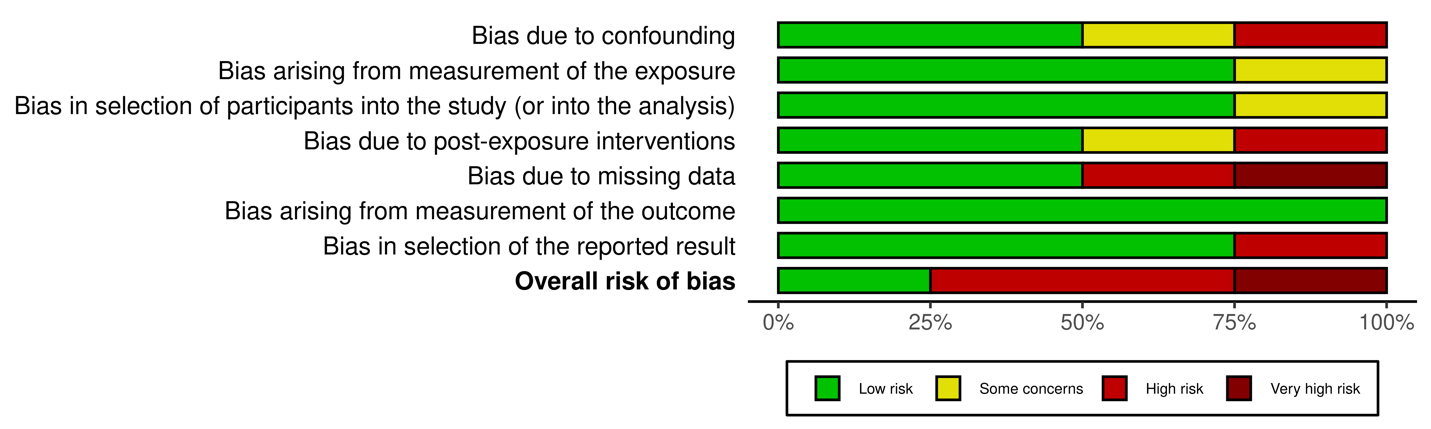


**Table S2. Outcomes of interest.**

| **Study ID** | **Title** | **Outcomes** | **Results of Interest** |
| --- | --- | --- | --- |
| Kirkham et al., 2021 | Weekday 16:8 Time-Restricted Eating in Breast Cancer Survivors: Feasibility, Safety, and Effects on Cardiometabolic Health | Feasibility, safety, visceral adipose tissue, | 8 weeks of TRE decreased the absolute CVD risk by a median of 2% and heart age by 6 years, and reversed the need for pharmacological treatment or metabolic syndrome in 45%. In all participants, visceral adipose tissue decreased by a median of 6% (p=0.018) and triglycerides by 17% (p=0.008). No significant changes in ectopic adipose tissue in the liver or thigh. |
| Vega et al., 2022 | Caloric restriction or intermittent fasting in cancer patients receiving curative radiotherapy | Adherence, anthropometric measures | Mean reduction in waist circumference of 4.9cm, of serum glucose 1.3 mg/dL, of triglycerides of 24.2 mg/dL and insulin of 2.1 $\mu$U/mL. |
| Badar et al., 2014 | Safety and Feasibility of Muslim Fasting While Receiving Chemotherapy | Safety, side effects | Statistically insignificant differences in means of: weight 1.04 95% CI -0.77m 2.85),  WBC (-0.30, 95% CI -3.87, 3.25), ANC (-0.14, 95% CI -2.89, 2.57), Hgb (- 0.09, 95% CI -0.75,0.55), Plt (-72.71 95% CI-143.5,-1.95), Cr (5.28 95% CI -2.54, 12.10), T.Bili (0.08 95% CI -3.75, 3.93), SGPT -3.8 95% CI -18.9, -11.2). 50% of patient report subjective improvements in fatigue. |
| Kirkham et al., 2022 | Time-Restricted Eating to Reduce Cardiovascular Risk Among Older Breast Cancer Survivors | Adherence, safety, efficacy, caloric intake | Change in: fat-free mass (−0.1 ± 1.6 kg; P = 0.76); mean MRI-derived VAT (−5% ± 7%; P = 0.009), median bioelectric-impedance derived whole-body fat mass (−0.9 kg; IQR: −1.5 to 0.1 kg; P = 0.046); median body mass decreased (−1.0 kg; IQR: −2.3 to 0.2 kg; P = 0.025); BMI (−0.2 ± 0.7 kg/m^2^; P = 0.10); median Framingham CVD risk decreased 15% relative change (P= 0.037). Total cholesterol, HDL, and systolic BP did not significantly change. |
| O'Donnell et al., 2022 | Pilot study to assess prolonged overnight fasting in breast cancer survivors (longfast) | BMI, blood biomarkers, compliance, fatigue | Median within-participant decrease in BMI relative to baseline measurements of 0.38 kg/m^2^ (p=0.0072). No significant changes in blood biomarkers. Improvements in anxiety (p = 0.0007) at 6 wks and anxiety (p = 0.0141), depression (p = 0.0048), and fatigue (p = 0.0105) at 12 wks |
| Alshammari et al., 2022 | The Impact of Fasting the Holy Month of Ramadan on Colorectal Cancer Patients and Two Tumor Biomarkers: A Tertiary-Care Hospital Experience. | Fasting practices, Tumor biomarkers (CEA, LDH), bloodwork | CEA reduced 46.9%, LDH reduced 55.6%. No statistically significant changes in hgb, WBC, platelets, eGFR, alk phos, lactate dehydrogenase. |
| Yassin et al., 2021 | Effects of Intermittent Fasting on Response to Tyrosine Kinase Inhibitors (TKIs) in Patients With Chronic Myeloid Leukemia: An Outcome of European LeukemiaNet Project | CBC, CR-ABL1 transcript levels, disease progression | MMR was maintained during and after Ramadan based on the median BCR/ABL1 values; (median 0.05, IQR 0.003, 2.9), (median 0.04, IQR 0.004, 0.63), (median 0.03, IQR 0.006, 0.30), before, during, and after Ramadan, respectively. Reduced levels of WBCs, neutrophils, basophils and BCR-ABL after fasting but statistically insignificant (P > 0.05). Platelet, hemoglobin, basophils, and eosinophils appeared to have a similar trend in their values measured before, during and after intermittent fasting periods. |
| Palomar-Cros et al., 2021 | The Association of Nighttime Fasting Duration and Prostate Cancer Risk: Results from the Multicase-Control (MCC) Study in Spain | Prostate cancer risk | A more extended nightly fast was linked to a more potent reduction of prostate cancer risk (OR = 0.77, 95% CI 0.54 -1.07). Having breakfast after 8:30 AM was associated with a non-significant increase risk of prostate cancer (OR = 1.30, 95% CI 0.92 - 1.85) compared to having breakfast at 8:30 AM or before. |
| Marinac et al., 2016 | Prolonged Nightly Fasting and Breast Cancer Prognosis | Cancer recurrence, mortality, sleep duration, Glucoregulation, inflammation, obesity | Fasting fewer than 13 hrs per night compared to $\geq$13 hrs: on recurrence (HR 1.36; 95% CI, 1.05-1.76), cancer-specific mortality (HR 1.21; 95% CI, 0.91-1.60), all-cause mortality (HR 1.22; 95% CI, 0.95-1.56). Each 2-hr increase in nightly fasting duration was associated with a 0.37-mmol/mol lower HbA1c level ($\beta$= -0.37; 95% CI, -0.72 to -0.01) and more hours of sleep per night ($\beta$ = 0.20; 95% CI, 0.14-0.26). Nightly fasting duration was not associated with BMI. |
| Kleckner et al., 2022 | Time-restricted Eating to Address Cancer-related Fatigue among Cancer Survivors: A Single-arm Pilot Study | Adherence, fatigue, other symptoms, caloric intake, safety, patient experience | Fatigue scores improved 5.3 ± 8.1 points on the FACIT-F fatigue subscale (*p*<0.001, effect size [ES]=0.55), 30.6 ± 35.9 points for the FACIT-F total score (*p*<0.001, ES=0.50), and -1.0 ± 1.7 points on the BFI (*p*<0.001, ES=-0.58). |
| Christensen et al., 2022 | Rationale and Design of IMPACT-women: A randomized controlled trial of the effect of time-restricted eating, healthy eating, and reduced sedentary behavior on metabolic health during chemotherapy for early-stage breast cancer. | CVD risk, chemotherapy symptoms, QOL | N/A |
| NCT05722288 | A Randomized, Phase II Clinical Trial of Time-Restricted Eating Versus Nutritional Counseling in Cancer Patients Receiving Radiation or Chemoradiation to Evaluate Its Impact on Toxicity and Efficacy | dsDNA damage, completion rate, adverse events, QOL, accumulated gH2ax foci, oxidative DNA damage | N/A |
| NCT05327608 | Intermittent Fasting for Patients With HER2- Negative and ER/PR <10% Breast Cancer and Body Mass Index >= 25 Receiving Neoadjuvant Chemotherapy | Adherence | N/A |
| NCT05312255 | Improving Host Factors in Patients With Monoclonal Gammopathies | Immune cell subsets, gut microbiome, bone markers, body composition, stress, fatigue, anxiety, functional status, nutritional behaviours | N/A |
| NCT05083416 | Effect of Prolonged Nightly Fasting (PNF) on Immunotherapy Treatment Outcomes in Patients With Advanced Head and Neck Cancer (HNSCC)-Role of Gut Microbiome | Compliance, gut microbiome and metabolites | N/A |
| NCT05023967 | Time Restricted Eating And Metformin (TEAM) in Invasive Breast Cancer (IBC) or Ductal Carcinoma in Situ (DCIS). A Randomized, Phase IIb, Window of Opportunity Presurgical Trial. | Dose limiting toxicity, of CIP2A-PP2A-GSK3beta-MCL-1 axis, Ki67 in cancer tissue, M30 and phosphorylated S6, physiological distress, eating habits, adverse events, blood biomarkers, | N/A |
| NCT04708860 | Pilot Study of the Impact of a Combined Intermittent Fasting and Exercise Intervention on Metabolic Markers in Patients With Advanced, Hormone Receptor Positive Breast Cancer | Rate of enrollment, adherence, metabolic biomarkers, QOL, patient-reported outcome measures | N/A |
| NCT04691999 | EFFECT-BC: The EFFECT of Intermittent Fasting on Body Composition in Women With Breast Cancer | Adherence, body fat, | N/A |
| NCT04626843 | Feasibility Study of Intermittent Fasting in Chronic Lymphocytic Leukemia and Small Lymphocytic Lymphoma (CLL/SLL) Patients at BC Cancer- Victoria | Lymphocyte count, QOL, inflammation, metabolomic profiles, autophagy status, immune cell gene expression, gut microbiome | N/A |
| NCT04560439 | METFIT Program - A Pilot Study Exploring the Feasibility of a Diabetes Prevention Program (DPP)-Based Lifestyle Modification Intervention to Reduce Insulin Resistance in Breast Cancer Survivors | Fidelity, retention, insulin resistance, | N/A |
| NCT04288336 | A Prospective Pilot Study Evaluating the Feasibility of Daily, Long-Term Intermittent Fasting for Men on PSA Surveillance Following Radical Prostatectomy for Localized, High-Risk Prostate Cancer | Adherence, PSA | N/A |
| NCT03523377 | The OnFACT study is a pilot randomized controlled trial of prolonged overnight fasting among adult survivors of childhood cancer. | Completion rate, glucose metabolism | N/A |
| NCT05259410 | The Safety and Efficacy of Time Restricted Eating Alone or Combined the Mediterranean Diet During Chemotherapy for Breast Cancer | Feasibility, dose intensity, treatment side effects, QOL, body weight and composition, glucose and insulin. | N/A |
| NCT04783467 | Feasibility and Acceptability of Time Restricted Eating (TRE) Among Endometrial Cancer Patients: the TREND Study | Feasibility, fidelity, acceptability, blood pressure, BMI and waist circumference, bloodwork | N/A |
